# Growth Pattern of Clear Cell Renal Cell Carcinoma in Patients with Delayed Surgical Intervention: Fast Growth Rate Correlates with High Grade and May Result in Poor Prognosis

**DOI:** 10.1155/2015/598134

**Published:** 2015-09-03

**Authors:** Lei Zhang, Wenshi Yin, Lin Yao, Xuesong Li, Dong Fang, Da Ren, Zhongyuan Zhang, Yu Fan, Qun He, Weimin Ci, Zhisong He, Liqun Zhou

**Affiliations:** ^1^Department of Urology, Peking University First Hospital, Institute of Urology, Peking University, National Urological Cancer Center, Beijing 100034, China; ^2^Beijing Institute of Genomics, Chinese Academy of Sciences, Beijing 100101, China

## Abstract

*Objectives.* Previous studies revealed an unclear correlation between the growth rate of renal cell carcinoma (RCC) and tumor grade and did not focus on certain histological subtype. This report investigated the correlation between the growth rate and tumor grade in clear cell RCC (ccRCC).* Methods*. We reviewed 60 patients with 61 ccRCC confirmed by delayed surgeries after at least 12 months of active surveillance. The linear growth rate (LGR), volumetric growth rate (VGR), and volume doubling time (VDT) were calculated, and their correlations with clinicopathologic characteristics were analyzed.* Results*. The mean LGR, VGR, and VDT were 0.86 (range 0–4.74) cm/year, 20.96 (range 0.31–211.93) cm^3^/year, and 667 (range 33–3321) days, respectively. ccRCCs with high grade had greater LGR (*P* < 0.001) and VGR (*P* = 0.001) and lower VDT (*P* = 0.017) than ccRCCs with low grade. Grade (OR = 5.185, *P* = 0.004) was the only independent risk factor of LGR >0.5 cm/year, and grade (OR = 3.006, *P* = 0.046) and initial size (OR = 0.392, *P* = 0.004) were independent risk factors of VDT <1 year. Five patients developed metastasis after surgery with LGR >0.5 cm/yr altogether; of them, four had cancer-related death by the last follow-up.* Conclusions*. Fast growth rate of ccRCC is significantly correlated with high tumor grade and may result in poor prognosis, especially for those with LGR >0.5 cm/yr.

## 1. Introduction

Due to high surgical comorbidity or short life expectancy for certain patients, active surveillance (AS) for renal tumors is being applied selectively by urologists in clinical practice. Although the risk of metastasis progression during AS is only approximately 2% [1], there is no effective systemic therapy for RCC. Inability of identifying lethal RCC is the most major problem while performing AS at present. Growth rate of renal tumors is believed to be the main trigger for intervention during AS. Anyway, there is no evidence supporting that the growth rate of RCC during AS is related to its prognosis so far. Because of the limitation of small sample size, the lack of pathological diagnosis, the generally favorable prognosis of RCC, and the follow-up not long enough until the cancer-related death occurred, it is hard to directly figure out whether fast growth rate under AS is related to poor prognosis of RCC.

Tumor grade is one of the most powerful prognostic factors for RCC [2]. The median 5-year RCC-specific survival is 94%, 86%, 59%, and 31% in patients with Fuhrman grades I, II, III, and IV RCC, respectively [3]. Hence, if the fast growth rate of RCC during AS is correlated with high tumor grade, we could indirectly believe that fast growth rate of RCC during AS could result in poor prognosis.

Although a prospective study with biopsy before AS is appropriate to investigate the correlation between the growth rate of RCC and grade intuitively, biopsy has the weakness in grading renal tumors comparing with surgical specimens. Hence, a retrospective study enrolling patients receiving delayed surgeries after AS is the unique opportunity to resolve this problem. However, only a few articles on this subject are available [4, 5]. All the available studies included small sample size and did not focus on a certain histological subtype. The biological behavior of RCC is different by histological subtypes. Grade 1 clear cell RCC may grow faster than grade 2 papillary RCC. Hence, the correlation between growth rate and grade of RCC is still unclear. For example, our previous studies confirmed a significantly higher growth rate in grade 2 RCC compared with grade 1 RCC; however, the growth rate of grade 3 was not significantly different from that of grade 1 or 2 RCC [4].

Clear cell RCC (ccRCC) accounts for 70–80% of all RCCs and is characterized as the common aggressive behavior compared to other subtypes [6]. Our previous study demonstrated that the growth rate of RCC tended to correlate with the histologic subtype and that ccRCC tended to grow faster than papillary cell carcinoma [4]. Hence, understanding the growth behavior of ccRCC is the most beneficial among all the subtypes.

On basis of these thoughts, we expand sample size and further focused on ccRCC to investigate the correlation between growth rate and tumor grade by reviewing 61 patients who had received delayed surgeries after at least 12 months of AS for renal tumors that later were confirmed to be ccRCC pathologically. In addition, we report our experience of AS with long-term of follow-up after surgery and characterize the ccRCC with failure of cancer control after delayed surgery.

## 2. Patients and Methods

### 2.1. Patient Selection

We retrospectively reviewed the kidney cancer databases at the Institute of Urology, Peking University, to identify patients with renal masses treated by AS initially for at least 12 months between January 1990 and August 2014. A total of 90 patients with 91 renal tumors were included. Patients without delayed surgical treatment until the last follow-up were excluded, and only ccRCC cases confirmed by surgical pathology were included. Patients with Von Hippel-Lindau syndrome or history of hereditary RCC were excluded. A total of 60 patients with 61 ccRCCs were included in the analysis.

### 2.2. Imaging Examination and Measurement of Lesions

During the period of AS, CT or MRI was performed at least every 6 months. When possible, the measurements were performed based on the same technique. All the images were reviewed by a professional radiologist and a urologic oncologist. The tumor size was defined as the maximal diameter of the tumor recorded during each imaging procedure. The linear growth rate (LGR) of a tumor was defined as the mean growth rate of the maximal diameter on a series of 2-dimensional images. The tumor volume was calculated as described in a previous study [7]: if 3 dimensions were present, the formula 0.5326*xyz* was employed; if 2 dimensions were available, the formula 0.5326*xy*(*x* + *y*/2) was used; if only 1 dimension was reported, the formula for volume of a sphere 0.5236 × 3 was employed. The volumetric growth rate (VGR) was defined as the average change in tumor volume per year. In addition, the volume doubling time (VDT) was also calculated based on the Schwartz equation, as previously described [8]: VDT = (*T* − *T*_0_) × log⁡⁡2/log⁡⁡(*V*/*V*_0_) (*T*: the date of the final imaging procedure, *T*_0_: the date of the initial imaging procedure, *V*: the volume at the final imaging evaluation, and *V*_0_: the volume at the initial imaging evaluation).

### 2.3. Pathological Examinations

Due to tumor growth, obvious enhancement on CT, or metastatic lesion, delayed surgical intervention was performed on all patients at Peking University First Hospital after a mean of 39.5 months of AS. All surgical specimens were reviewed by two senior pathologists who were blinded to the patients' personal data. The pathological results confirmed ccRCC for all tumors. The histological classification was determined by the Heidelberg typing system. The tumor stage was assessed according to the 2002 American Joint Committee on Cancer TNM staging system, and tumor grading was performed according to the Fuhrman grade system.

### 2.4. Statistical Analysis

The chi-squared test was used to test the distribution of categorical variables. The correlations between two continuous variables were assessed by calculating Pearson's correlation coefficient. The Mann-Whitney *U* or Kruskal-Wallis *H* test was used to compare two or three groups of continuous variables. A logistic regression analysis was used to identify the independent risk factors of an LGR <0.5 cm/year and a VDT <1 year. The SPSS v.14.0 software package (SPSS Inc., Chicago, IL, USA) was used for data processing. *P* < 0.05 was considered statistically significant.

## 3. Results and Discussion

### 3.1. AS of ccRCC

A total of 60 patients with 61 ccRCCs treated by delayed treatment after at least 12 months of AS were identified for analysis. The clinical, demographic, and tumor characteristics are summarized in Table 1. Of the 61 tumors, 49 (80.3%) were asymptomatic for the entire clinical course and diagnosed incidentally during imaging procedures for physical examination; the other 12 cases had complaints of flank pain or occasional hematuria at presentation. The reasons that patients initially choose AS included patient preference (41 of 60, 68.3%), a benign diagnosis considered at presentation (15 of 60, 25.0%), the existence of bilateral disease (2 of 60, 3.3%), and concomitant malignancy (2 of 60, 3.3%). Of the 60 patients, 48 (78.7%) were male, and 12 (21.3%) were female. The mean patient age was 55 years (range, 26–81).

**Figure 1:**
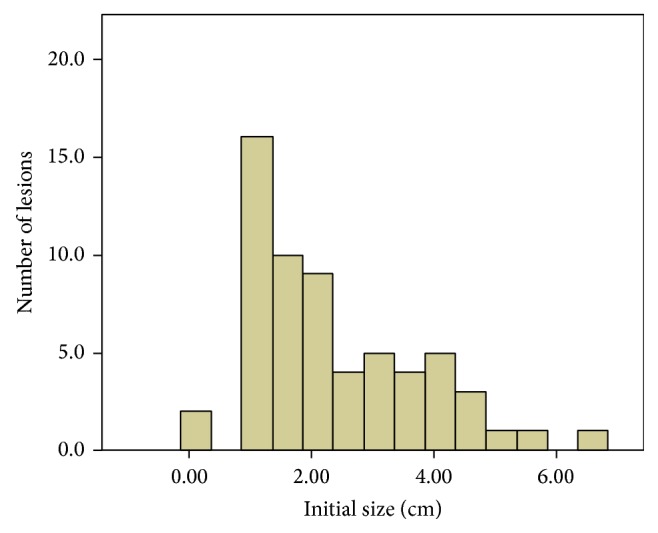
Distribution of initial tumor sizes of ccRCCs.

After a mean AS of 39.5 months, the mean tumor size increased from 2.32 cm (range, 0.10–6.70) at presentation to 4.44 cm (range, 1.40–11.80). The distribution of tumor size is shown in Figure 1; of 52 (85.2%) tumors, the initial tumor size was ≤4 cm at presentation. Stage progression was documented in 22 tumors: 15 tumors progressed from T1a to T1b, 6 tumors progressed from T1a to T2, and 1 tumor progressed from T1b to T2. No disparity between pT stage and cT stage at operation was found for any tumor. Only one patient (1.7%) developed metastatic disease during AS. The patient presented with a biopsy-proven metastasis ccRCC in the lung at the 155th month of AS; during this period, the primary tumor size increased from 1.6 cm to 4.4 cm. Although this tumor did not grow fast, the mean LGR was 0.20 cm/year.

The indication of surgical intervention included tumor growth, the presence of obvious enhancement on CT, or metastatic lesion (palliative excision of the primary lesion). Forty-four of the 61 tumors (72.1%) were treated by radical nephrectomy; the other 17 (27.9%) tumors were treated by partial nephrectomy. The pathological results confirmed ccRCC in all 61 tumors. Thirteen tumors (21.3%) were grade 1, 38 tumors (62.3%) were grade 2, and 10 tumors (16.4%) were grade 3.

### 3.2. Growth Kinetics, the Correlation between Growth Rate and Tumor Grade

The results of LGR, VGR, and VDT and their association with clinicopathologic variables are summarized in Table 2. The mean of LGR, VGR, and VDT was 0.86 (range, 0–4.74) cm/year, 20.96 (range, 0.31–211.93) cm^3^/year, and 667 (range, 33–3321) days, respectively. ccRCC with a high grade had a greater LGR (*P* < 0.001) and VGR (*P* = 0.001) and a lower VDT (*P* = 0.017) than ccRCC with a low grade. High-grade ccRCC showed significantly aggressive growth kinetics compared to low-grade ccRCC.

**Figure 2:**
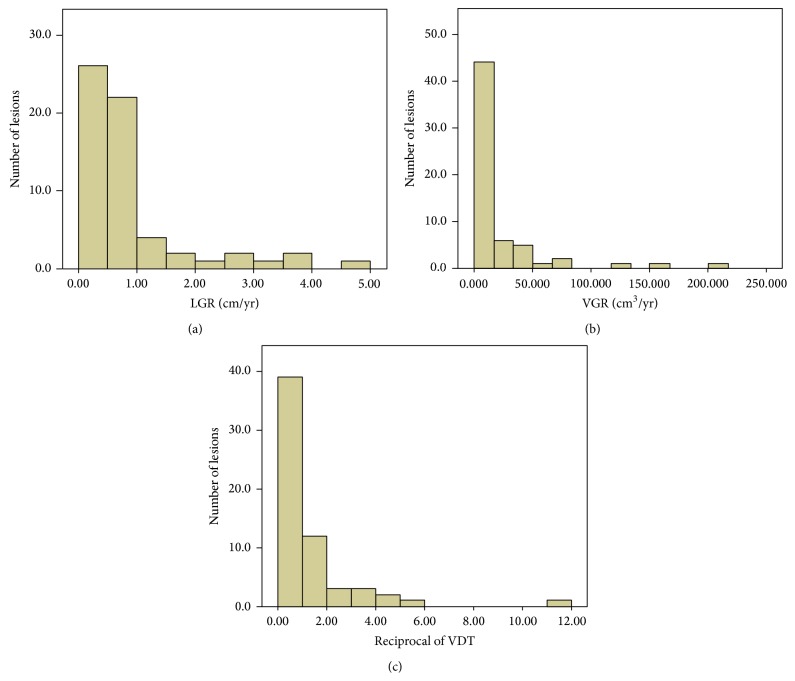
(a) Distribution of linear growth rate (LGR, cm/year) of ccRCCs. (b) Distribution of the volumetric growth rate (VGR, cm^3^/year) of ccRCCs. (c) Distribution of the reciprocal of the volume doubling time (VDT) (calculated as 365 divided by VDT) of ccRCCs.

The distribution of LGR and VGR for ccRCC is shown in Figures 2(a)-2(b). Thirty-four (55.7%) ccRCCs presented an LGR >0.5 cm/year, and the other 27 ccRCCs showed slow growth, with an LGR ≤ 0.5 cm/year; only one (1.6%) ccRCC showed zero growth in maximal diameter during 17 months of AS. According to the patients' age, sex, and initial tumor size, no correlations with LGR or VGR were found (*P* > 0.05). A logistic regression analysis revealed that tumor grade (OR = 5.185, *P* = 0.004) was the only independent risk factor of an LGR >0.5 cm/year for ccRCC.

The distribution of reciprocal of VDT (calculated as 365 divided by VDT) is shown in Figure 2(c). For 22 (36.1%) ccRCCs, VDT was less than 1 year, and 10 ccRCCs revealed a VDT <0.5 years. VDT was weakly positively correlated with the initial tumor size (*r* = 0.335, *P* = 0.008): ccRCC with a smaller initial size had a shorter VDT than ccRCC with a larger initial size. No correlation between the patients' age or sex and VDT was found. The logistic regression analysis revealed that tumor grade (OR = 3.006, *P* = 0.046) and initial size (OR = 0.392, *P* = 0.004) were independent risk factors of a VDT <1 year for ccRCC.

### 3.3. Postoperative Follow-Up

Six patients (9.8%) were lost during follow-up after surgery. Regarding the remaining 55 patients, the median follow-up after surgery was 50 months. Of them, 5 patients developed metastasis after surgery: 1 case of lung metastasis, 1 case of brain metastasis, 1 case of pleural and pulmonary metastasis, 1 case of metastasis in the neck, and 1 case of metastasis in the head of the pancreas. The data of the 5 patients were summarized in Table 4. The LGR values were all greater than 0.5 cm/year in the 5 patients. The tumors sizes were larger than 5 cm at operation for 4 patients of them. In the entire cohort, 5 deaths were found after a mean of 25.8 months of follow-up after surgery; of them, 4 were cancer-related deaths, and the remaining one death was related to cardiovascular events.

### 3.4. Discussion

As most renal masses are removed surgically soon after detection, it is difficult to characterize the natural history of RCC. However, AS is becoming gradually accepted, especially for patients with a high risk of surgery and limited life expectancy, providing a unique opportunity for understanding the natural history of RCC. Consistent with the aggressive features of ccRCC, previous studies [4, 9] have demonstrated that ccRCC shows a trend of rapid growth compared with other subtypes of RCC. A pooled analysis demonstrated that renal tumors that progressed during AS were predominantly ccRCC [1]. It would be expected that a complete understanding of ccRCC growth pattern during AS may help in selecting optimal patients for AS in consideration of the risk of fast-growing tumors in such cases.

We also reviewed past reports about the natural history of renal masses and performed a pooled analysis (Table 3) involving 1171 patients with 1271 renal tumors [4, 5, 7, 8, 10–27]. Based on this pooled analysis, we found that the renal masses generally grew slowly and seldom metastasized. The initial tumor size ranged from 1.73 to 7.2 cm. The mean age ranged from 52.2 to 80.4 years. The means of LGR and VGR were 0.33 (range, 0.06–0.8) cm/year and 11.0 cm^3^/year, respectively. Compared with LGR, VGR is rarely used to describe the growth kinetics of renal masses. Only 19 (1.6%) patients developed metastatic disease during AS. Although a lack of pathological result is a major limitation for previous studies, of the 1271 renal tumors in the present study, only 444 (34.9%) had pathological results, and 380 (29.9%) were RCC. No work has reported the growth kinetics of ccRCC in detail, with the rarity of samples and lack of pathological results being the major reason for scarce knowledge about the growth pattern of ccRCC.

Based on the pooled analysis, the current study revealed a larger LGR (0.86 cm/yr versus 0.33 cm/yr) and VGR (20.96 cm^3^/yr versus 9.48 cm^3^/yr) and a younger age (55 years versus 69.5 years). Although Kouba and colleagues reported that younger patients show a faster growth rate than older patients [17], we did not find a significant correlation between age and growth rate in ccRCC. Only inclusion of cases with delayed surgical treatment might bring bias of growth rate, because the tumors that need delayed surgeries after AS usually have rapid growth rate. Anyway, to eliminate the bias, we further focused on the studies where all the cases were treated by delayed surgery and confirmed to be RCC pathologically, four studies with 96 cases were enrolled (Table 3) [4, 5, 8, 12], and the LGR of our cohort is still greater (0.86 cm/yr versus 0.62 cm/yr). However, all the four studies did not focus on certain pathological subtype to discuss the growth rate. These results suggested that ccRCC might have a rapid growing potential among the whole of RCC. Hence, the fast growth kinetics in the present study is partly due to the presence of ccRCC pathology for all cases.

Inability of identifying the lethal RCC is the most concern when applying AS. The growth rate of renal tumors is the most common observational index during AS, and renal tumors would be excised if showing rapid growth during AS. Although a pooled analysis on small renal masses progressing to metastases under AS revealed that renal masses with metastatic progression have a relatively rapid growth compared to those without metastasis during AS [1], there is no definitive evidence demonstrating that a rapid growth rate is an independent risk factor of poor prognosis. It is hard to make clear the correlation between the growth rate of RCC during AS and prognosis, because it needs a large number of samples, pathological diagnosis, and the long enough follow-up until the cancer-related death occurs; however, RCC has a favorable prognosis generally. It is widely accepted that tumor grade is correlated with the prognosis of RCC, and high tumor grade of RCC indicates a poor prognosis [3]. In a compromise way, if it could be proved that fast growth rate of RCC during AS correlates with high tumor grade, we believe that fast growth rate of RCC during AS may result in poor prognosis. So it is essential to make clear the correlation between the growth rate of RCC during AS and tumor grade.

As percutaneous renal biopsy is unreliable for small tumors [28] and may underestimate tumor grade of RCC [29], reviewing the patients with renal tumors which received delayed surgery and hence got the pathological diagnosis with grade and histological type is the only opportunity to investigate the correlation between the growth rate of RCC during AS and tumor grade. At present, the correlation between tumor grade and the growth rate of RCC is still controversial. Kato and colleagues demonstrated a significantly higher LGR in RCCs with grade 3 compared with RCCs with grade 2 (*P* = 0.01); however, they did not observe a significant difference in growth rate between grades 1 and 2 and between grades 1 and 3 [5]. Our previous studies also confirmed a significantly higher LGR in grade 2 RCC compared with grade 1 RCC, and the LGR of grade 3 tended to be faster than that of grades 1 and 2 RCC; however, the difference was not significant [4]. These studies suggested a correlation between RCC grade and growth rate, but the correlation remains unclear. It should be noted that these studies did not focus on certain histological subtype when discussing the correlation between grade and growth rate in RCC. In the current study, when focusing on ccRCC, we found a clear and strong significant correlation that the growth rate of ccRCC with a higher grade was faster than that of ccRCC with a lower grade, regardless of the measurement index used (i.e., LGR, VGR, or VDT). Additionally, tumor grade was found to be an independent risk factor of an LGR <0.5 and a VDT <1 year in ccRCC. Our findings more precisely reflect the correlation between grade and growth rate in ccRCC.

In the present study, we did not find that the initial tumor size correlated with LGR or VGR; however, we did find a positive correlation between initial tumor size and VDT: ccRCC with a smaller tumor size has a shorter VDT compared with ccRCC with a larger tumor size. Consistent with our results, Crispen and colleagues demonstrated that smaller renal tumors grew faster than larger renal tumors [22]. These results suggest Gompertzian growth kinetics in ccRCC, which theorizes that the growth rate of tumors is exponential initially and decreases with an increase in tumor size.

There were 5 patients who developed metastatic disease after surgery; 4 of them have died of RCC by the last follow-up. The ccRCC in these patients showed rapid growth kinetics that the LGR were all greater than 0.5 cm/year. In addition, the tumor sizes exceed 5 cm at operation for 4 of the 5 patients. Up to now, there is no definite indication of surgical treatment during AS. Jewett et al. had recommended treatment if the tumor grew rapidly or reached 4 cm in maximal diameter [25]. Based on our results, we recommend treatment for patients with renal masses during AS if the tumor size reaches 4 cm in maximal diameter or the LGR reaches 0.5 cm/year. However, these criteria still need to be validated.

## 4. Conclusions

We discovered a strongly significant correlation between the growth rate of ccRCC during AS and tumor grade. Fast growth rate during AS for ccRCC correlates with high tumor grade and may result in a poor prognosis, especially for ccRCCs with LGR >0.5 cm/yr. AS should be used cautiously for ccRCC with a fast growth rate. Further investigation on the natural history of non-ccRCC subtypes is needed. More attention should be paid to identify the lethal RCC for early intervention.

## Figures and Tables

**Table 1:** Patient demographics and tumor characteristics.

Sex	
Men (%)	48 (78.7)
Women (%)	13 (21.3)
Age, yr	
Median	56
Mean	55
Range	26–81
Side	
Left (%)	32 (52.5)
Right (%)	29 (47.5)
Initial tumor size	
Maximal diameter, cm	
Median	1.90
Mean	2.32
Range	0.10–6.70
Volume, cm^3^	
Median	3.591
Mean	11.186
Range	0.001–82.967
Final tumor size	
Maximal diameter, cm	
Median	4.00
Mean	4.44
Range	1.40–11.8
Volume, cm^3^	
Median	28.595
Mean	66.992
Range	0.982–560.017
Duration of AS, mo	
Median	27.00
Mean	39.5
Range	12–155
Grade	
1	13
2	38
3	10
Pathological stage	
T1a	30
T1b	20
T2	6
T3	5

AS: active surveillance; LGR: linear growth rate; DT: doubling time.

**Table 2:** Growth rate for ccRCC: overall and correlation with clinicopathologic variables.

	LGR (cm/yr)	VGR (cm^3^/yr)	VDT (days)
Overall growth per year, *n* = 61			
Median	0.61	7.49	561
Mean ± SD	0.86	20.96	667
Range	0.00–4.74	0.31–211.93	33–3321
Growth rates and clinicopathologic variables			
Grade			
1, *n* = 13	0.32 ± 0.06	6.10 ± 3.15	885.69 ± 169.00
2, *n* = 38	0.74 ± 0.11	12.14 ± 2.27	684.08 ± 107.79
3, *n* = 10	2.03 ± 0.50	73.79 ± 22.79	319.60 ± 87.29
*P* value	<0.001^*^	0.001^*^	0.017^*^
Age			
*R*	−0.061	0.001	−0.045
*P* value	0.638	0.991	0.733
Initial size			
*R*	0.207	0.027	0.335
*P* value	0.110	0.836	0.008^*^
Sex			
Men, *n* = 48 (mean ± SD)	0.83 ± 0.14	20.15 ± 6.05	661.15 ± 91.29
Women, *n* = 13 (mean ± SD)	1.00 ± 0.28	23.95 ± 6.47	690.00 ± 169.95
*P* value	0.355	0.098	0.673

LGR: linear growth rate; VGR: volumetric growth rate; VDT: volume doubling time.

^*^Statistically significant.

**Table 3:** Published series on the natural history of renal masses.

	Year	Patients/lesions (*n*)	Mean age (years)	Mean initial MTD (cm)	Mean follow-up (months)	Mean LGR (cm/year)	Mean VGR (cm^3^/year)	Progression to metastasis, *n* (%)	Pathologic RCC
Fujimoto et al. [10]	1995	6/6	59.7	2.47	24	0.47	9.7	0 (0)	5/5
Bosniak et al. [11]	1995	37/40	65.5	1.73	39	0.36	5.26	0 (0)	22/26
Oda et al. [12]	2003	16/16	54^a^	2.0^a^	25	0.54^a^	—	0 (0)	16/16^*^
Volpe et al. [7]	2004	29/32	71^a^	2.48	27.9	0.1	3.8	0 (0)	8/9
Wehle et al. [13]	2004	29/29	70	1.83	32	0.12	—	0 (0)	3/4
Kato et al. [5]	2004	18/18	56.5	2.0	27	0.42	4.4	0 (0)	18/18^*^
Lamb et al. [14]	2004	36/36	76.1	7.2	27.7	0.39	—	1 (2.8)	23/24
Chawla et al. [15]	2006	49/61	71	2.97	36	0.2	—	1 (1.6)	16/21
Youssif et al. [16]	2007	35/44	71.8	2.2	47.6	0.21	2.7	2 (5.7)	6/8
Kouba et al. [17]	2007	43/46	67	2.92	32.8	0.7	—	0 (0)	12/14
Siu et al. [18]	2007	41/47	68	2.0	29	0.27	—	1 (2.4)	10/16
Fernando et al. [19]	2007	13/13	80.4	5.01	38.38	0.17	11.97	1 (7.7)	0
Matsuzaki et al. [20]	2007	15/15	67	2.2	38	0.06	0.67	0 (0)	3/3
Lee et al. [8]	2008	30/30	65.5	2.6	12.6	0.59	19.1	3 (10.0)	30/30^*^
Beisland et al. [21]	2009	63/65	76.3	4.3	33	0.66	—	2 (3.2)	15/18
Crispen et al. [22]	2009	154/173	69	2.45	31	0.285	17.0	2 (1.3)	52/61
Rosales et al. [23]	2010	212/223	71^a^	2.8^a^	35^a^	0.34^a^	—	4 (1.9)	32/40
Hwang et al. [24]	2010	56/58	64.3	2.1	22	0.21	1.9	0 (0)	10/15
Jewett et al. [25]	2011	127/151	73	2.1	28	0.13	—	1 (0.7)	37/46
Li et al. [4]	2012	32/32	52.2	2.14	46	0.8	—	0 (0)	32/32^*^
Mehrazin et al. [26]	2014	68/72	68.9	5.3	38.9	0.44	—	0 (0)	16/23
Brunocilla et al. [27]	2014	62/64	75	2.0	91.5	0.4	4.6	1 (1.6)	14/16

Total		1171/1271	69.5	2.82	34.6	0.33	9.48	19 (1.6)	380/444
This study		60/61	55	2.32	39.46	0.86	20.96	1 (1.6)	61/61

^a^Median.

—: not stated

^∗^All cases received delayed surgical intervention and were confirmed to be renal cell carcinoma (RCC) pathologically.

**Table 4:** Clinical and pathological characteristics of SRMs that progressed to metastasis after delayed treatment.

Cases	Sex	Age (years)	Grade	ITS (cm)	UTS (cm)	LGR (cm/year)	Duration of AS (months)	Surgical treatment	Time to metastasis after surgery	Site of metastasis	Outcome
1	Male	63	3	6.7	8.8	0.9	28	RN	9	Pleura and lung	Mortality at 57 mo. after surgery
2	Male	61	2	4.5	5.4	1.08	10	RN	16	Neck	Mortality at 24 mo. after surgery
3	Female	65	1	1.9	2.81	0.61	18	RN	14	Lung	Alive at 20 mo. after surgery
4	Male	58	3	0.1	8.0	4.74	20	RN	12	Brain	Mortality at 19 mo. after surgery
5	Female	59	2	3.6	7.0	1.28	32	PN	66	Head of pancreas	Alive at 101 mo. after surgery

SRMs: small renal masses; ITS: initial tumor size; UTS: ultimate tumor size; LGR: linear growth rate; RN: radical nephrectomy; PN: partial nephrectomy.
